# Unexpected Spinal Epidural Abscess: A Real Emergency

**DOI:** 10.7759/cureus.75013

**Published:** 2024-12-03

**Authors:** Beatriz R Marques, Mariana Certal, Elisabete Cerqueira, Mariana Macedo, Beatriz Exposito

**Affiliations:** 1 Internal Medicine, Unidade Local de Saúde de Trás-os-Montes e Alto Douro, Chaves, PRT; 2 Internal Medicine, Unidade Local de Saúde de Trás-os-Montes e Alto Douro, Vila Real, PRT; 3 Nephrology, Unidade Local de Saúde de Trás-os-Montes e Alto Douro, Vila Real, PRT; 4 Internal Medicine, Centro Hospitalar Trás-os-Montes e Alto Douro (CHTMAD), Chaves, PRT

**Keywords:** bacterial infection, compressive myelopathy, crural tetraparesis, diagnostic delay, progressive myelopathy, spinal epidural abscess, staphyloccocus aureus

## Abstract

Cervical and lower back pain are classic reasons for patients to seek care in the emergency department (ED). However, in rare instances, they signal serious underlying conditions, posing a significant diagnostic challenge. A 72-year-old male with history of lumbar spine surgery many years ago presented to the ED with neck pain for the last five days as well as bilateral lower limb weakness and feet paresthesia. His neurologic deficits rapidly progressed to crural tetraparesis, with a sensitive abnormal level by C4, associated with urinary retention and fever. An urgent cervical and dorsal magnetic resonance imaging (MRI) was performed, revealing an anterior fluid collection, causing secondary medullar compression, consistent with spinal epidural abscess (SEA). Cervical epidural abscess is an uncommon but potentially life-threatening condition that can lead to swift and irreversible neurological deterioration. With this clinical case, the authors highlight the relevance of clinical suspicion, interdisciplinary and coordinated work in the diagnosis of epidural abscess, as a time-dependent emergency.

## Introduction

Back pain, either lower back or cervical pain are highly prevalent and classic reasons for patients to seek care in the emergency department (ED) [1,2]. Frequently they often stem from self-limiting, low-risk conditions, such as musculoskeletal conditions making imaging studies unnecessary as they expose patients to avoidable radiation and increase healthcare costs. However, specifically neck pain can be caused by more severe spinal injuries or even non-spinal causes for instance tension headache, carotid dissection or even coronary artery disease.

As a diagnostic challenge, the physician has to be aware of all signs, symptoms and red flags so as not to leave some potentially severe disease untreated.

The authors present an example of a frequent misdiagnosis in the ED to alert physicians to a condition that can leave permanent sequelae if not treated in time.

## Case presentation

Patient is a 72-year-old male, emigrant in France, former plumber, with history of lumbar spine surgery many years ago. He did not report any known medical diseases and did not take any medication. Two weeks prior to admission he came to the ED complaining of back pain and was discharged with pain medication. Two weeks later he returned to the ED with neck pain for the last five days as well as unspecific discomfort. He also reported bilateral lower limb weakness and feet paresthesia. When finally observed by the internal medicine team he could not get up or raise his arms and claimed stiffness in all four limbs. On a more thorough examination, he presented with a crural tetraparesis, with a muscular power scale of G2 on the right superior limb and G3 on the others. On a sensitive level a mild hypoesthesia was present, by C4 level, associated with limb “electric shocks”. Reflexes globally reduced and Kernig or Brudzinski signs were absent. During his ED stay he developed urinary retention needing vesical catheterization. He presented with high fever (39ºC), arterial pressure 140/77 mmHg, and a heart rate 100 bpm.

Initial blood work showed a mild thrombocytopenia, normal renal function, elevated inflammatory markers, with C reactive protein (CRP) of 27.6 mg/dL. No anemia or abnormal coagulation parameters were present. Urine analysis was innocent (Table 1).

**Table 1:** Analytic study at admission. aPTT - activated Partial Thromboplastin Time; ALT - Alanine Transaminase; AST - Aspartate Transaminase; INR - International Normalised Ratio; γ-GT - gamma-Glutamyl Transferase.

Parameter	Result	Reference Value
Hemoglobin	14.3	12-16 g/dL
Leucocyte count	10.86	4-11 x 10^3^/uL
Neutrophil count	9.490	1.5-8.0 x 10^3^/uL
Platelets	126.000	150-400 x 10^3^/uL
Urea/creatinine	25/0.8	<50/0.5-0.9 mg/dL
Sodium/Potassium	139/3.8	135-147/3.7-5.1 mEq/L
AST	25	<35 U/L
ALT	51	<33 U/L
γ-GT	81	7-32 U/L
Alkaline Phosphatase	113	35-105 U/L
Total Bilirubin	0.8	<1.2 mg/dL
Lactate Dehydrogenase	198	135-214 U/L
INR	1.03	<1.2
aPTT	29.7	27-38 seg
C Reactive Protein	27.6	<0.5 mg/dL
Urine sediment	Normal	-
Respiratory virus panel	Negative	-

**Figure 1:**
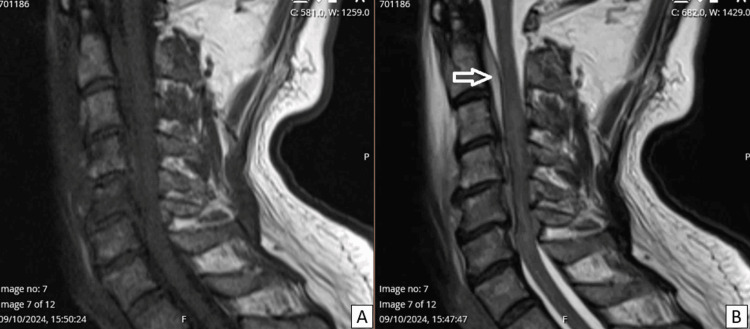
(A) Sagittal T1- and (B) T2-weighted MRI. Spinal epidural abscess is noted anterior do the medulla with high signal intensity (arrow). This inflammation continues to the pre-vertebral compartment.

He was first submitted to a cervical and neck computed tomography (CT) which showed some unspecific inflammatory changes on the anterior section of the vertebral canal from C4 to C7. After consultation with a neurosurgeon, an urgent cervical and dorsal magnetic resonance imaging (MRI) was performed, revealing an anterior fluid collection, causing secondary medullar compression, consistent with spinal epidural abscess (SEA) (Figure 1). He was quickly transferred to the nearest neurosurgical center and submitted to an urgent cervical laminectomy C4, C5 and C6 and drainage of the abscess.

As an inpatient, he was started on broad-spectrum antibiotics with vancomycin, ceftazidime and metronidazole. The initial blood cultures were positive for methicillin-susceptible Staphylococcus aureus (MSSA), as well as the second blood cultures obtained five days after admission. The pus collected from surgery unfortunately did not have any results. Antibiotic therapy was de-escalated to intravenous flucloxacillin carried out over three weeks and switched to oral for another three weeks as recommended by the infectious diseases team.

**Figure 2:**
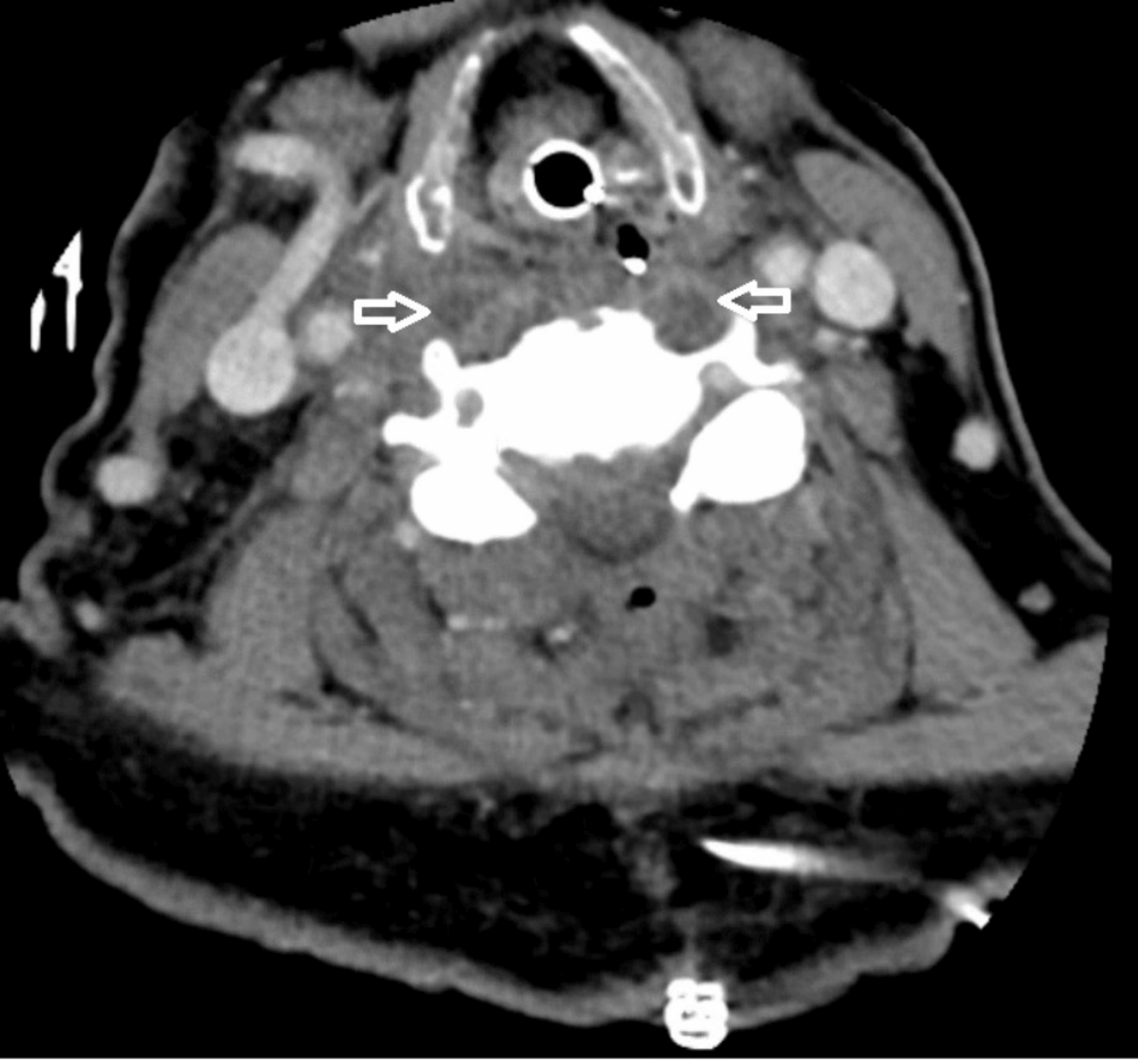
Cervical computed tomography showing pre-vertebral abscess (arrows).

As for the search for the cause of this SEA, a transthoracic echocardiography did not show any signs of vegetations. A post-surgery cervical CT showed proper medullar decompression but also signs of pre-vertebral abscesses (Figure 2). After consultation with otorhinolaryngology, best medical therapy was suggested. The patient was also observed by a stomatology specialist which still provided no clues regarding the source of the infection.

After a favorable control cervical MRI, the patient was discharged after one month of admission and nearly three weeks of physical therapy, highly improved from his neurological deficits and managing to walk with bilateral support.

## Discussion

SEA is defined as a collection of purulent material in the spinal canal's epidural space. Although relatively rare, recent data suggests that SEA’s hospitalizations have increased to nearly one in 1000 maybe due to a higher number of immunocompromised individuals, increased amount of invasive spinal surgeries or even higher detection rate with MRI [3,4].

The most common clinical manifestation is lower back pain, as the most prevalent localization is the lumbar spine. In fact, the patient presented earlier initially complained of lower back pain but with no neurological symptoms or fever.

Cervical SEA represents only 19% of all SEAs and is a more urgent condition as the smaller epidural space in the cervical region tolerates less inflammation and because of the vulnerability of the atlantoaxial joint. Additionally, spinal cord compression in this area can impair breathing by affecting diaphragmatic innervation from C3, C4, and C5 [5].

The nonspecific presentation of SEA makes its diagnosis unexpected and particularly challenging. Not every physician does a thorough neurological exam to every back or neck pain or even performs blood tests. Diagnostic delays are common, ranging from 75% to 89% [6,7], with patients often seen by multiple physicians before a diagnosis is established [8]. In this case, prior lumbar spine surgery should have raised some suspicion to other diagnoses.

The classic symptom triad of SEA includes back pain, fever, and neurological deficits. However, few patients exhibit all three at presentation, as seen in this case. Cervical epidural abscesses typically cause localized cervical pain or stiffness, though symptoms can also manifest as nonspecific or radiating pain to the head or lower back. A thorough neurological examination, including cranial nerves, and airway inspection are essential to assess for sensorimotor deficits. However, a normal neurological exam does not rule out SEA [9,10].

Identifying risk factors can help in the early establishment of the diagnosis. Common risk factors include diabetes, immunosuppression, obesity, trauma, spinal anesthesia, epidural catheter placement, intravenous drug use, and prior spinal instrumentation [9]. In this case, the patient only referred to a lumbar spine intervention many years ago, with no other known risk factor.

The infection reaches the epidural space through hematogenous spread, contiguous spread from vertebral osteomyelitis, for example, or direct inoculation from spinal procedures. However, in many patients, no clear source of infection is identified [10]. In this case, the epidural abscess likely arose from the contiguous spread of a pre-vertebral abscess. The exact source of the prevertebral infection was unclear.

MRI remains the gold standard for diagnosing SEA due to its high sensitivity (up to 95%) and specificity (over 90%) [9,10]. Inflammatory markers, while nonspecific, are supportive in the diagnostic process. Blood and cerebrospinal cultures may not always identify the causative pathogen, though Staphylococcus aureus is the most commonly isolated organism in SEA cases [5,10].

Based on current evidence, surgical decompression and abscess drainage, followed by culture-guided intravenous antibiotics, is the preferred treatment approach and was crucial in achieving a favorable outcome for this patient [10]. Although no randomized clinical trials have defined the optimal treatment duration for SEA, a course of four to eight weeks is typically recommended [10,11]. Prompt surgical intervention is particularly critical for cervical abscesses, patients showing neurological symptoms, or those with worsening inflammatory markers [5].

## Conclusions

Cervical and lower back pain are typically associated with low-risk conditions that do not require radiological evaluation; however, in rare cases, they may indicate a serious underlying disease. Identifying such pathologies can be particularly challenging in the absence of red flags, needing a high level of clinical suspicion. Assessing for neurological deficits is crucial, and rapid imaging should be performed when such deficits are detected.

Cervical epidural abscess is an uncommon but potentially life-threatening condition that can lead to swift and irreversible neurological deterioration. With this clinical case, the authors highlight the relevance of interdisciplinary and coordinated work in the diagnosis of epidural abscess, as a time-dependent emergency.
